# Impact of home- and center- based physical training program on cardio-metabolic health and IGF-1 level in elderly women

**DOI:** 10.1186/s11556-019-0220-7

**Published:** 2019-08-08

**Authors:** Dora Praksch, Barbara Sandor, David Kovacs, Peter Petrovics, Krisztina Kovacs, Kalman Toth, Eszter Szabados

**Affiliations:** 1 First Department of Medicine, Division of Cardiology and Angiology, University of Pécs, Medical School, Pécs, Hungary; 2 First Department of Medicine, Division of Preventive Cardiology and Rehabilitation, University of Pécs, Medical School, Rákóczi Street 2, Pécs, H-7624 Hungary; 3 Department of Biochemistry and Medical Chemistry, University of Pécs, Medical School, Pécs, Hungary

**Keywords:** Physical activity, IGF-1, Elderly female patients, Cardio-metabolic health

## Abstract

**Background:**

Data in the literature concerning the effects of physical activity on lipid and IGF-1levels are controversial in postmenopausal women. The aim of the present study was to determine the combined effects of a 12 weeks home-based walking program aiming to achieve 10,000 steps daily and a center- based aerobic exercise training on functional capacity, some important cardio-metabolic parameters, IGF-1 level and psychological items among elderly female patients. Sixty female patients (67.4 ± 5 years) with moderate to high cardiovascular risk were randomly assigned either to an exercise training program for 12 weeks or to the control group.

**Results:**

Our organized training program resulted in a significantly improved daily physical activity (4232 [IQR: 3162–7219] to 8455 [IQR: 6757–11,488]; *p* < 0.001 ft-steps), functional capacity (MET) (8.17 ± 1.57 to 8.87 ± 1.76) (*p* = 0.002), metabolic status including total cholesterol (5.17 ± 1.13 to 4.77 ± 1.12 mmol/l), LDL cholesterol (3.37 ± 1.05 to 2.81 ± 0.98 mmol/l), triglyceride (1.68 ± 0.71 to 1.28 ± 0.71 mmol/l) and HgbA1c (6.24 ± 0.67 to 6.06 ± 0.58 mmol/l), as well as IGF-1 (59.68 ± 27.37 to 66.79 ± 22.74 ng/ml) levels (*p* < 0.05) in the training group. From psychological tests only physical functionality improved significantly (*p* = 0.03) in the training group. The training group significantly differed from the control group in four parameters including MET (*p* = 0.003), LDL-cholesterol (*p* = 0.046), triglyceride (*p* = 0.001) and IGF-1 levels (*p* < 0.001) after the intervention.

**Conclusion:**

The applied home-, and- center based training program effectively increased the daily physical activity of the elderly female patients and improved several cardio-metabolic parameters. Further investigations are needed on larger patient population to establish our findings and examine how these positive changes may decrease CV events and mortality.

## Background

Regular physical activity (PA) is widely recommended throughout the human lifespan to maintain health and physical fitness [1–3]. Menopause is a critical state in the life of women generally accompanied by dysregulation in the cardio-metabolic profile resulted from critical changes in body composition such as excessive accumulation of fat at visceral level. Increase of physical activity level could modulate these negative changes both in body composition and cardio-metabolic profile. Elderly women who are physically active possess less risk of functional limitations and a higher health-related quality of life [4, 5]. Furthermore, osteoporosis, sarcopenia, risk of falls [6, 7], dementia, depression, loss in cognitive function [6], and the risk of some type of cancers [8] can be reduced by regular PA. Part of the cardio-metabolic health including physical performance, systolic/diastolic blood pressure, resting heart rate, fasting levels of plasma glucose and insulin level, abdominal visceral adipose tissue, weight, BMI, sedentary behavior are also positively influenced by PA in elderly women [3, 6, 9–12]. However its effect on certain metabolic parameters, such as lipid levels are not unequivocal. Some investigations have proved that PA favorably modifies lipid parameters among elderly women [9, 13], while others failed to demonstrate significant effect [10, 14–17].

Insulin-like growth factor 1 (IGF-1) is a basic peptide composed of 70 amino acids, which is thought to play a central role in metabolism [18], cancer development [19], CV diseases [20] and aging [21]. In adults high levels of IGF-1 are associated with increased cancer risk [22] and CV diseases. A population-based study examining the association of different IGF-1 levels with mortality, cardiovascular disease, and cancer in the elderly has found a U-shaped relationship between IGF-1 level and fatal CV diseases, which means that both high and low levels of IGF-1 were associated with increased risk of CV mortality. Significant associations of serum IGF-1 with fatal or non-fatal cancer were not observed in this elderly population [23]. Serum IGF-1 level is declining with age [24] and postmenopausal women generally display even lower levels of IGF-1 compared to elderly men [21] which may in part explains increased CV mortality in postmenopausal women. Low levels of IGF-1 are associated with osteoporosis [25], disability [26], neurodegenerative illnesses such as Alzheimer dementia and brain atrophy [27] and increased risk of CVD [28]. The anti-inflammatory and anti-oxidant effects of higher IGF-1 level on blood vessels have also been investigated [29]. The development of impaired glucose tolerance and type 2 diabetes is also more expected in patients with low IGF-1 levels [24, 30]. Regular PA has several health preserving effects and it has been examined previously how it may modulate IGF-1 level. Some investigations have demonstrated positive effect of especially resistance training on IGF-1 levels [31–34], while aerobic exercise training had no considerable effect on IGF-1 concentrations [33, 35, 36].

Elderly women usually do not report only physical but also psychological and social changes during menopause that affect their global and CV health [37]. General psychological-, and emotional well-being, and optimism are related to health promoting behaviors including healthy eating and lifestyle habits and self-care, supporting CV and overall health of elderly patients [38–40].

The aim of our study was to investigate whether 12 weeks of an applied home- and center- based physical training program is sufficient to change functional capacity, some important cardio-metabolic parameters, IGF-1 level and psychological items of elderly female patients with moderate to high CV risk.

## Methods

### Ethics approval and consent to participate

The investigation was approved by the Regional Ethics Committee of the University of Pécs (No. 5829) and was conducted in accordance with the ethical principles stated in the Declaration of Helsinki. A written informed consent was obtained from all subjects.

### Patients

Sixty female non-smoker patients with moderate to high CV risk (mean age: 67.4 ± 5 years) were enrolled into our study (Table 1). Patients were recruited either from primary care or from internal medicine and cardiology outpatient care by different physicians. They voluntarily agreed to participate in the study and then were randomly assigned either to the CV preventive training program or to the control group. Participants in both groups met the following inclusion criteria: ejection fraction (EF) ≥55% and metabolic equivalent (MET) ≥5. Exclusion criteria were the following: previous CV events, heart failure, inducible myocardial ischemia and arrhythmias on an exercise stress test. Medication and drug therapy were not modified during the study in either groups (Table 2). It was also suggested to the control group not to change their usual physical activity level in the next 12 weeks.

**Table 1:** Characteristics of the study population, *n* = 60

population characteristic	training group (*n* = 30)	control group (*n* = 30)	*p* value
hypertension	29 (96%)	27 (90%)	0.30
diabetes mellitus	10 (33%)	9 (30%)	0.78
dyslipidemia	19 (63%)	15 (50%)	0.29
chronic kidney disease	2 (3.3%)	0 (0%)	0.15

**Table 2:** Medication therapy during the 12 week training program, *n* = 60

medication	training group (*n* = 30)	control group (*n* = 30)	*p* value
statins	19 (63%)	15 (50%)	0.29
antiplatelet drugs	9 (30%)	7 (23%)	0.56
β-blocker	15 (50%)	14 (46%)	0.79
RAAS inhibitor	20 (66%)	14 (46%)	0.12
calcium channel blocker	8 (26%)	9 (30%)	0.77
antidiabetic drugs	10 (33%)	9 (30%)	0.78
diuretics	12 (40%)	8 (26%)	0.27

RAAS renin–angiotensin–aldosterone system

### Study design

At baseline patients were examined using electrocardiography (ECG) and echocardiography to exclude unknown cardiac problems that could limit their ability to exercise. Then they were tested on treadmill according to the Bruce protocol to assess functional capacity. The intensity of the training was defined as 50–70% of the peak maximal oxygen consumption (VO2max), starting at 50% and gradually increased to 70%. Metabolic laboratory such as fasting glucose, hemoglobin A1c (HgbA1c), total cholesterol (TC), low density lipoprotein (LDL) cholesterol, high density lipoprotein (HDL) cholesterol, triglyceride (TG), IGF-1 measurements and SF-36 (36-Item Short Form Survey) Questionnaire were performed. Upon reaching week 12, all tests were repeated, with the exception of echocardiography.

### Home-based walking program

A daily walking program was implemented, which could be performed in a 10–15 min workout and could be completed by the patients solely on their own. For appropriate estimation of the daily walking program our patients were asked to wear a personalized activity tracker on their wrist [41]. These trackers did not only registered the daily footsteps but also motivated our elderly women to achieve the daily activity goal of 10,000 footsteps based on health expert’s recommendation [42, 43].

### Aerobic exercise training program

The aerobic exercise training program began with warm-up exercises (breathing exercises and stretching of the large joints) for 5–10 min three times weekly. In the second phase, patients participated in a moderate-intensity training. The training involved static (exercises with a medicine ball, half-squats, toe raises and body flexions) and dynamic (walking, jogging, ball games e.g., basketball, football) exercise elements. The intensity of the training was defined as 50–70% of the peak maximal oxygen consumption (VO_2_max), starting at 50% and gradually increased to 70%. The aerobic phase lasted 35–40 min. Finally, relaxation exercises were performed (stretching and breathing exercises) for 10 min. The exercise training was supervised by a cardiologist and conducted by a physiotherapist. Pulse and blood pressure were taken prior to, during (20 min after starting the training) and at the end of the training period.

### Blood collecting

At baseline and after 12 weeks, blood samples were obtained from the antecubital vein in both groups. The blood was collected into one clot activator-coated and gel-containing (5 ml), one potassium EDTA-coated (3 ml) and one sodium fluoride and potassium oxalate-coated (2 ml) Vacutainer tubes were sent for laboratory measurements and one potassium EDTA-coated (3 ml) Vacutainer tube was sent for IGF-1 measurements.

### IGF-1measurements

IGF-1 levels were measured using Human IGF-1 Quantikine ELISA Kit (R&D Systems; Cat. No.: RD-DG100). EDTA-plasma samples were collected from patients at the beginning and after the 12th week, the samples were stored at − 74 °C until performing the assay. The assay employs a quantitative sandwich immunoassay technique. The IGF-1 assay protocol was carried out according to the manufacturer’s instructions.

### Psychological surveys

SF-36 Questionnaire was applied to examine the psychological effects of the 12 week home - and center based training program on the perception of health. It is a self-administered questionnaire measuring health over 8 dimensions (vitality, physical functioning, bodily pain, general health perceptions, physical role functioning, emotional role functioning, social role functioning, mental health). Both the training- and the control group rate their health status on a scale from 0 (worst health) to 100 (best health).

### Statistical analysis

A sample size and power analysis was performed for the overall population using PASS software. For the sample size of *n* = 28 patients (1:1 enrollment ratio of interventional and control group) needed to detect a true difference of d = 2 in MET levels with 95% power, where type I error probability is α = 0.05.

Statistical analysis was performed using the IBM SPSS statistical software version 23. Data were shown as mean ± standard deviation (SD). Significance level was defined as *p* < 0.05. To check differences in the interventional and in the control group we performed dependent-t test. For testing how the two groups varied in time the interaction of time x group effect was applied. The normality was analyzed by Kolmogorov-Smirnov test. All the studied parameters in both groups showed no significant deviation from a normal distribution (*p* > 0,05; df:56). The nonparametric Wilcoxon Rank test was applied to analyze potential changes in psychological functioning and in the number of foot-steps, since these were ordinal variables. Data were shown as median and IQR.

## Results - within groups

### Home-based walking and center based physical training program increased patients’ exercise capacity and improved metabolic parameters

**Fig. 1:**
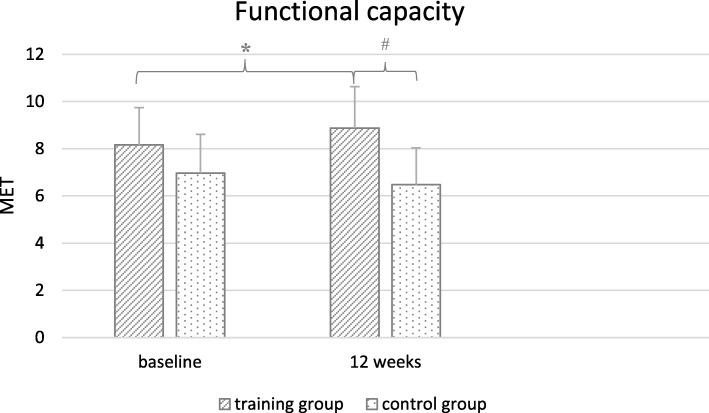
Significant changes in metabolic equivalent (MET) within and between the groups. Values are shown baseline and 12 weeks (mean ± SD). Levels of significance *p* < 0.005. *: *p* = 0.002 regarding baseline to 12 weeks in the training group; #: *p* < 0.01 regarding the training group compare to the control group after 12 weeks

Home-based walking program resulted in a significant improvement in daily physical activity (4232 [IQR: 3162–7219] vs 8455 [IQR: 6757–11,488] foot-steps) among our female patients (*p* < 0.001). We did not register any adverse events during the trial. The combined home-based and center based physical training program improved exercise capacity, described by the significantly increased metabolic equivalent (MET) in the training group (*p* = 0.002) (Fig. 1). Exercise capacity did not change in the control group.

Total cholesterol, LDL cholesterol, TG, and HgbA1c level indicated a significant decrease during the investigated period (*p* < 0.05), the other measured laboratory parameters did not show significant changes in the training group (Table 3). None of the laboratory parameters changed in the control group.

**Table 3:** Changes in metabolic laboratory parameters and IGF-1 level after 12 weeks physical activity in the training group. *N* = 30; values are baseline and 12 weeks means±SD. Levels of significance: *p* < 0.05

measured parameters	baseline	12 week	*p* value
HgbA_1C_ (mmol/l)	6.24 ± 0.67	6.06 ± 0.58	0.007
total cholesterol (mmol/l)	5.17 ± 1.13	4.77 ± 1.12	0.042
LDL-cholesterol (mmol/l)	3.37 ± 1.05	2.81 ± 0.98	0.003
HDL-cholesterol (mmol/l)	1.46 ± 0.39	1.51 ± 0.46	ns
triglycerides (mmol/l)	1.68 ± 0.71	1.28 ± 0.71	0.002
IGF-1 (ng/ml)	59.68 ± 27.37	66.79 ± 22.74	0.006

Body weight (BW) and body mass index (BMI) differed neither in the training group, nor in the control group after 12 weeks (data are not shown).

### Home-based walking and center-based physical training program increased IGF-1 level

Serum IGF-1 significantly increased after 12 weeks in the training group (Table 3), while it decreased in the control group (*p* < 0.05).

### Home-based walking and center-based physical training program increased patients’ physical functioning

Participants of the training group reported significantly fewer limitations in their everyday physical functioning (p < 0.05), however in the other examined psychological items no significant change could be observed following the training program (data are not shown). Participants in the control group did not report any changes in their psychological conditions (data are not shown).

## Results – between groups

### Home-based walking and center-based physical training program increased patients’ exercise capacity and IGF-1 level, and improved lipid parameters

**Fig. 2:**
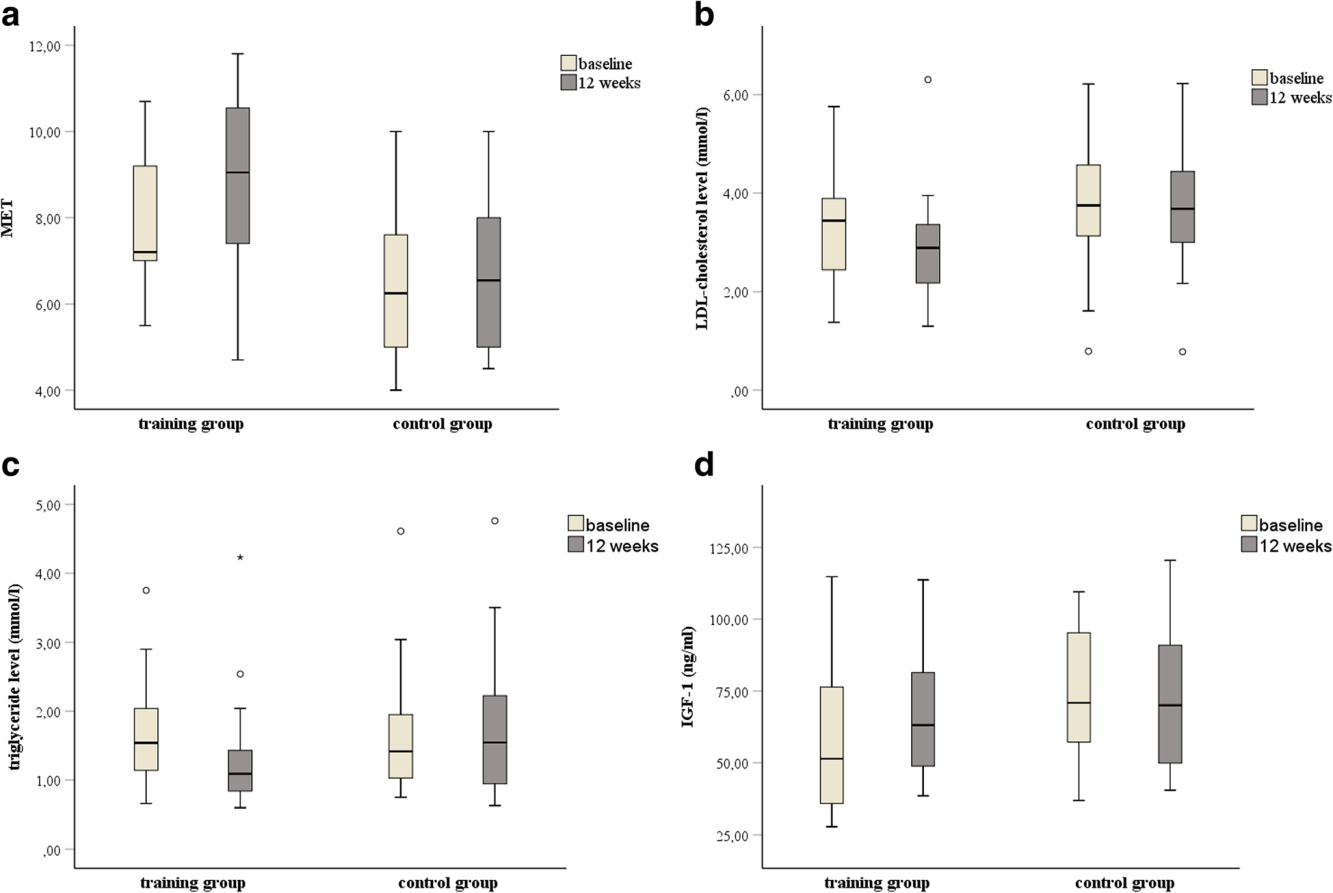
Box plots of cardio-metabolic parameters and IGF-1 level at baseline and after 12 weeks between the training-, and the control group. n = 60. Levels of significance: p < 0.05. **a** Significant difference in MET between the groups. *p* = 0.003. **b** Significant difference in LDL-cholesterol level between the groups. *p* = 0.046. **c** Significant difference in triglyceride level between the groups. *p* = 0.001. **d** Significant difference in IGF-1 level between the groups. *p* < 0.001

The interaction of time x group effect revealed, that the training group significantly differed from the control group in four parameters including MET (*p* = 0.003) (Fig. 2a), LDL-cholesterol (*p* = 0.046) (Fig. 2b), triglyceride (*p* = 0.001) (Fig. 2c) and IGF-1 levels (*p* < 0.001) (Fig. 2d) after the intervention.

The training group did not differ from the control group in the other investigated cardio-metabolic parameters (total-cholesterol-, HDL-cholesterol-, and HgbA1c level) after the training program (data are not shown).

### Home-based walking and center-based physical training program and psychological status

Psychological testing did not show significant differences between the training group and the control group (data are not shown).

## Discussion

In our present study we investigated the effects of a home-, and center based physical training program on functional capacity, metabolic laboratory, IGF-1 levels and psychological parameters in elderly female patients with moderate to high CV risk. The organized training program resulted in a significantly improved functional capacity, metabolic status including LDL cholesterol, triglyceride, HgbA1c and IGF-1 level, and physical functionality.

Maintenance of a physically active lifestyle is a great challenge especially for the elderly population [4] and women are less likely to adhere physical training programs compared to men [44]. We assumed that a combined, home-based walking and a center based training program fits well to the everyday life of the elderly ladies, and a center-based exercise program led by a physiotherapist might be effective and enjoyable for this special patient population. Walking is a low cost and easy way of PA for the elderly [45]. Most of the studies reported that the normal daily activity of healthy adults is only 4000 to 6000 steps [46, 47] and in older women it is even lower [48, 49]. Although in our study the elderly female patients could not completely fulfill the daily target of 10,000 steps, still the achieved significant improvement in daily PA (4232 [IQR: 3162–7219] to 8455 [IQR: 6757–11,488] footsteps) is a great performance taking into accounts their age and co-morbidities.

After 12 weeks of the home- and center- based physical training program, we could demonstrate an average of 0.7 MET improvement in functional capacity (8.17 ± 1.57 to 8.87 ± 1.76). According to data in the literature an increase by 1 MET in cardiorespiratory fitness could reduce the risk of all causes and CV mortality by 13 and 15%, respectively [50]. Furthermore, the training group significantly differed from the control group in MET level after the intervention, suggesting that the training program significantly improved the functional capacity of our elderly female patients.

In our study we observed a significant decrease in the total cholesterol as well as in LDL cholesterol and TG levels in the training group, while in the control group no change could be observed in the metabolic parameters. In addition, the training group significantly differed from the control group in LDL cholesterol and triglyceride levels after the training program, referring that the observed favorable changes are due to the home- and center-based training program. Data in the literature regarding the effects of PA on lipid levels in general populations and also in elderly females is controversial. Examining the reasons behind this phenomenon we found some interesting observations. Fonong et al. reported that 2 months regular leisure time activity in elderly woman and men is too short to induce changes in body composition and plasma lipid levels [16]. Nieman et al. could not demonstrate changes in HDL-cholesterol after 12 weeks cardiorespiratory exercise in previously sedentary elderly women. They indicated that women tend to have higher HDL-cholesterol level than men, furthermore it is harder to increase the already higher HDL-cholesterol level, and PA mostly has more favorable effects on young or middle aged than elderly women [17]. Di Blasio et al. failed to report improvement in lipid levels after 13 week moderate intensity exercise program among postmenopausal women. They observed a decrease in spontaneous daily PA during the training program which may negatively affected the efficiency of the program [10]. On the other hand, Fahlman et al. demonstrated favorable changes in plasma lipoprotein profile after 10 weeks endurance or resistance training among elderly women, although LDL-cholesterol level decreased significantly only in the resistance training group [13]. Kemmler et al. reported decreased plasma lipid levels after 26 months intense exercise program among postmenopausal women [9]. Examining our and the above described different training programs we may realize that those physical training programs were able to induce significant changes in lipid levels in elderly females which either contained resistance training [13] or was intense and long enough [9] or could effectively increase the daily physical activity level, like the present home- and center- based exercise program.

It was previously demonstrated that regular PA improves plasma glucose level as well as plasma insulin concentration [10, 11, 51]. In accordance with previous studies following the home- and center- based training program HgbA1c significantly decreased among our elderly female patients, contributing significantly to the positive metabolic effects of PA. In the control group no change could be observed in the HgbA1C level.

At baseline low levels of IGF-1 were measured in our study (Table 3). In the training group significant increase in IGF-1 levels could be observed but still remained below the average level of healthy middle-aged female adults [52]. Moreover, the training group differed significantly from the control group in IGF-1 levels after the intervention, suggesting that the home- and center-based training program caused the beneficial changes in the IGF-1 levels. It is known that IGF-1 level markedly declines with aging which is also referred to as somatopause and this could be more robust around the time of menopause [53, 54]. It has been previously proved that resistance training improved IGF-1 levels in healthy adults [31], elderly males [32], patients with sarcopenic obesity [33], and also in postmenopausal women [34]. No association has been previously reported between aerobic PA levels and IGF-1 concentrations in postmenopausal women [33, 35, 36]. However, in a large cross-sectional study the effect of physical activity on hormone levels were examined among the postmenopausal women, a more intense PA estimated by the Cambridge Index was associated with higher IGF-1 concentrations [55]. Based on previous results and our findings it seems that in the case of aerobic exercise training a more intensive PA level is needed to change IGF-1 level. The decrease in IGF-1 levels in the control group may be due to the lack of regular PA.

Besides objective measurements SF36 questionnaire was applied in our study to measure the psychological well-being of our elderly female patients. The physical functionality, which is the patients’ subjective judgement of their physical state has been improved after our home-, and center- based training program, meaning they have experienced fewer limitations during their everyday physical tasks, like shopping, walking or bathing. This better physical functionality was in accordance with the improved functional capacity measured by treadmill. However, no significant improvement could be measured in other examined psychological parameters. A longer follow up period may be necessary for achieving significant changes in other psychological parameters. A previous study examining 6 month exercise training in postmenopausal women attenuated the unfavorable psychological changes associated with menopause [56].

Our study indicated, that elderly women with moderate to high CV risk were able to achieve the level of physical activity necessary to result in favorable changes in cardio-metabolic profile and IGF-1 level. The subjective perception of their physical performance has also changed positively.

### Study limitation

Participating in an exercise intervention cannot be blinded which means a general limitation in these types of investigations. Moreover our study group was relatively small, so further measurements with a larger population are needed to substantiate our findings.

## Conclusion

The present study demonstrated a significant improvement in several cardio-metabolic parameters such as functional capacity, physical functioning, total as well as LDL cholesterol, TG, HgbA1c and IGF-1 levels of elderly female patients with moderate to high CV risk after 12 weeks of home- and center-based training program.

Achieving significant changes in IGF-1 and lipid levels by a physical training program seems to be more difficult than in the case of other cardio-metabolic parameters. According to our findings and data in the literature in order to improve IGF-1 level and lipid parameters in elderly women physical training programs should either contain resistance training elements or be intensive enough or effectively increase the daily physical activity level.

## Data Availability

The datasets used and/or analyzed during the current study are available from the corresponding author on reasonable request.

## Abbreviations

CV: Cardiovascular
CVD: Cardiovascular disease
ECG: Electrocardiography
EF: Ejection fraction
HDL: High density lipoprotein
IGF-1: Insulin-like growth factor 1
LDL: Low density lipoprotein
MET: Metabolic equivalent
PA: Physical activity
SD: standard deviation
SF-36: 36-Item Short Form Survey
TC: Total cholesterol
Vo_2_max: Peak maximal oxygen consumption

## References

[1] Piepoli MF, Hoes AW, Agewall S, Albus C, Brotons C, Catapano AL (2016). 2016 European guidelines on cardiovascular disease prevention in clinical practice: the sixth joint task force of the European Society of Cardiology and Other Societies on cardiovascular disease prevention in clinical practice. Eur Heart J.

[2] Vanhees L, De Sutter J, Gelada SN, Doyle F, Prescott E, Cornelissen V, Kouidi E, Dugmore D, Vanuzzo D, Börjesson M, Doherty P (2012). Importance of characteristics and modalities of physical activity and exercise in the management of cardiovascular health in individuals with cardiovascular risk factors: recommendations from the EACPR. Part II Eur J Prev Cardiol.

[3] Church TS, Earnest CP, Skinner JS, Blair SN (2007). Effects of different doses of physical activity on cardiorespiratory fitness among sedentary, overweight or obese postmenopausal women with elevated blood pressure: a randomized controlled trial. JAMA..

[4] Buchner DM (2009). Physical activity and prevention of cardiovascular disease in older adults. Clin Geriatr Med.

[5] Bergland A, Fougner M, Lund A, Debesay J (2018). Ageing and exercise: building body capital in old age. Eur Rev Aging Phys Act.

[6] Mendoza N, De Teresa C, Cano A, Godoy D, Hita-Contreras F, Lapotka M, Llaneza P, Manonelles P, Martínez-Amat A, Ocón O, Rodríguez-Alcalá L, Vélez M, Sánchez-Borrego R (2016). Benefits of physical exercise in postmenopausal women. Maturitas..

[7] Hita-Contreras F, Martínez-Amat A, Cruz-Díaz D, Pérez-López FR (2016). Fall prevention in postmenopausal women: the role of Pilates exercise training. Climacteric..

[8] Eliassen AH, Hankinson SE, Rosner B, Holmes MD, Willett WC (2010). Physical activity and risk of breast cancer among postmenopausal women. Arch Intern Med.

[9] Kemmler W, Lauber D, Weineck J, Hensen J, Kalender W, Engelke K (2004). Benefits of 2 years of intense exercise on bone density, physical fitness, and blood lipids in early postmenopausal osteopenic women: results of the Erlangen fitness osteoporosis prevention study (EFOPS). Arch Intern Med.

[10] Di Blasio A, Ripari P, Bucci I, Di Donato F, Izzicupo P, D'Angelo E, Di Nenno B, Taglieri M, Napolitano G (2012). Walking training in postmenopause: effects on both spontaneous physical activity and training-induced body adaptations. Menopause..

[11] Mandrup CM, Egelund J, Nyberg M, Lundberg Slingsby MH, Andersen CB, Løgstrup S, Bangsbo J, Suetta C, Stallknecht B, Hellsten Y (2017). Effects of high-intensity training on cardiovascular risk factors in premenopausal and postmenopausal women. Am J Obstet Gynecol.

[12] Nilsson A, Wåhlin-Larsson B, Kadi F (2017). Physical activity and not sedentary time per se influences on clustered metabolic risk in elderly community-dwelling women. PLoS One.

[13] Fahlman MM, Boardley D, Lambert CP, Flynn MG (2002). Effects of endurance training and resistance training on plasma lipoprotein profiles in elderly women. J Gerontol A Biol Sci Med Sci.

[14] Danielson ME, Cauley JA, Rohay JM (1993). Physical activity and its association with plasma lipids and lipoproteins in elderly women. Ann Epidemiol.

[15] Shigematsu R, Tanaka K, Nho H, Nakagaichi M, Takeda M, Tomita T, Unno H, Ohkawa S (2000). Effects of exercise conditioning on vital age in hyperlipidemic women. J Physiol Anthropol Appl Hum Sci.

[16] Fonong T, Toth MJ, Ades PA, Katzel LI, Calles-Escandon J, Poehlman ET (1996). Relationship between physical activity and HDL-cholesterol in healthy older men and women: a cross-sectional and exercise intervention study. Atherosclerosis..

[17] Nieman DC, Warren BJ, O'Donnell KA, Dotson RG, Butterworth DE, Henson DA (1993). Physical activity and serum lipids and lipoproteins in elderly women. J Am Geriatr Soc.

[18] Oh KJ, Lee DS, Kim WK, Han BS, Lee SC, Bae KH. Metabolic Adaptation in Obesity and Type II Diabetes: Myokines, Adipokines and Hepatokines. Int J Mol Sci. 2016;18(1):pii: E8. 10.3390/ijms18010008 PMC5297643 28025491

[19] Renehan AG, Zwahlen M, Minder C, O'Dwyer ST, Shalet SM, Egger M (2004). Insulin-like growth factor (IGF)-1, IGF binding protein-3, and cancer risk: systematic review and meta regression analysis. Lancet.

[20] Juul A, Scheike T, Davidsen M, Gyllenborg J, Jorgensen T (2002). Low serum insulin-like growth factor I is associated with increased risk of ischemic heart disease: a population-based case–control study. Circulation.

[21] O'Connor KG, Tobin JD, Harman SM, Plato CC, Roy TA, Sherman SS, Blackman MR (1998). Serum levels of insulin-like growth factor-1 are related to age and not to body composition in healthy women and men. J Gerontol A Biol Sci Med Sci.

[22] Christopoulos PF, Msaouel P, Koutsilieris M (2015). The role of the insulin-like growth factor-1 system in breast cancer. Mol Cancer.

[23] van Bunderen CC, van Nieuwpoort IC, van Schoor NM (2010). The Association of Serum Insulin-like Growth Factor-I with mortality, cardiovascular disease, and Cancer in the elderly: a population-based study. J Clin Endocrinol Metab.

[24] LeRoith D, Yakar S (2007). Mechanisms of disease: metabolic effects of growth hormone and insulin-like growth factor 1. Nat Clin Pract Endocrinol Metab.

[25] Hamrick MW, McNeil PL, Patterson SL (2011). A role for Myokines in muscle-bone interactions. Exerc Sport Sci Rev.

[26] Cappola AR, Bandeen-Roche K, Wand GS, Volpato S, Fried LP (2001). Association of IGF-I levels with muscle strength and mobility in older women. J Clin Endocrinol Metab.

[27] Westwood AJ, Beiser A, Decarli C, Harris TB, Chen TC, He XM, Seshadri S (2014). Insulin-like growth factor-1 and risk of Alzheimer dementia and brain atrophy. Neurology..

[28] Boger RH, Frystyk J, Ledet T, Moller N, Flyvbjerg A, Orskov H. Low serum insulin-like growth factor I is associated with increased risk of ischemic heart disease. Circulation. 2003;27: 107(20):e193. 10.1161/01.CIR.0000074249.75310.65 12777324

[29] Higashi Y, Quevedo HC, Tiwari S, Sukhanov S, Shai SY, Anwar A, Delafontaine P (2014). Interaction between insulin-like growth factor-1 and atherosclerosis and vascular aging. Front Horm Res.

[30] Thankamony A, Capalbo D, Marcovecchio ML, Sleigh A, Jørgensen SW, Hill NR, Dunger DB (2014). Low circulating levels of IGF-1 in healthy adults are associated with reduced β-cell function, increased intramyocellular lipid, and enhanced fat utilization during fasting. J Clin Endocrinol Metab.

[31] Borst SE, De Hoyos DV, Garzarella L, Vincent K, Pollock BH, Lowenthal DT, Pollock ML (2001). Effects of resistance training on insulin-like growth factor-I and IGF binding proteins. Med Sci Sports Exerc.

[32] Tsai CL, Wang CH, Pan CY, Chen FC (2015). The effects of long-term resistance exercise on the relationship between neurocognitive performance and GH, IGF-1, and homocysteine levels in the elderly. Front Behav Neurosci.

[33] Chen HT, Chung YC, Chen YJ, Ho SY, Wu HJ (2017). Effects of different types of exercise on body composition, muscle strength, and IGF-1 in the elderly with Sarcopenic obesity. J Am Geriatr Soc.

[34] Orsatti FL, Nahas EA, Maesta N, Nahas-Neto J, Burini RC (2008). Plasma hormones, muscle mass and strength in resistance-trained postmenopausal women. Maturitas..

[35] Vitiello MV, Wilkinson CW, Merriam GR, Moe KE, Prinz PN, Ralph DD, Colasurdo EA, Schwartz RS (1997). Successful 6-month endurance training does not alter insulin-like growth factor-I in healthy older men and women. J Gerontol A Biol Sci Med Sci.

[36] Poehlman ET, Rosen CJ, Copeland KC (1994). The influence of endurance training on insulin-like growth factor-1 in older individuals. Metabolism..

[37] Kumari M, Stafford M, Marmot M (2005). The menopausal transition was associated in a prospective study with decreased health functioning in women who report menopausal symptoms. J Clin Epidemiol.

[38] Giltay EJ, Geleijnse JM, Zitman FG, Buijsse B, Kromhout D (2007). Lifestyle and dietary correlates of dispositional optimism in men: the Zutphen elderly study. J Psychosom Res.

[39] Steptoe A, Gibson EL, Hamer M, Wardle J (2007). Neuroendocrine and cardiovascular correlates of positive affect measured by ecological momentary assessment and by questionnaire. Psychoneuroendocrinology..

[40] Ostir GV, Markides KS, Black SA, Goodwin JS (2000). Emotional well-being predicts subsequent functional independence and survival. J Am Geriatr Soc.

[41] Lohne-Seiler H, Hansen BH, Kolle E, Anderssen SA (2014). Accelerometer-determined physical activity and self-reported health in a population of older adults (65-85 years): a cross-sectional study. BMC Public Health.

[42] Hatano Y (1993). Use of the pedometer for promoting daily walking exercise. ICHPER.

[43] Yamanouchi K, Takashi T, Chikada K, Nishikawa T, Ito K, Shimizu S, Ozawa N, Suzuki Y, Maeno H, Kato K, Oshida Y, Sato Y (1995). Daily walking combined with diet therapy is a useful means for obese NIDDM patients not only to reduce body weight but also to improve insulin sensitivity. Diabetes Care.

[44] Grace SL, Midence L, Oh P, Brister S, Chessex C, Arthur HM (2016). Cardiac rehabilitation program adherence and functional capacity among women: a randomized controlled trial. Mayo Clin Proc.

[45] McElrath M, Myers J, Chan K, Fonda H (2017). Exercise adherence in the elderly: experience with abdominal aortic aneurysm simple treatment and prevention. J Vasc Nurs.

[46] Choi BC, Pak AW, Choi JC, Choi EC (2007). Achieving the daily step goal of 10.000 steps: the experience of a Canadian family attached to pedometers. Clin Invest Med.

[47] Tudor-Locke C, Ainsworth BE, Whitt MC, Thompson RW, Addy CL, Jones DA (2001). The relationship between pedometer-determined ambulatory activity and body composition variables. Int J Obes Relat Metab Disord.

[48] Conn VS, Burks KJ, Minor MA, Mehr DR (2003). Randomized trial of 2 interventions to increase older women’s exercise. Am J Health Behav.

[49] Jensen GL, Roy MA, Buchanan AE, Berg MB (2004). Weight loss intervention for obese older women: improvements in performance and function. Obes Res.

[50] Kodama S, Saito K, Tanaka S, Maki M, Yachi Y, Asumi M, Sone H (2009). Cardiorespiratory fitness as a quantitative predictor of all-cause mortality and cardiovascular events in healthy men and women: a meta-analysis. JAMA..

[51] Rossen J, Yngve A, Hagströmer M, Brismar K, Ainsworth BE, Iskull C, Möller P, Johansson UB (2015). Physical activity promotion in the primary care setting in pre- and type 2 diabetes - the Sophia step study, an RCT. BMC Public Health.

[52] Bidlingmaier M, Friedrich N, Emeny RT, Spranger J, Wolthers OD, Roswall J (2014). Reference intervals for insulin-like growth factor-1 (igf-i) from birth to senescence: results from a multicenter study using a new automated chemiluminescence IGF-I immunoassay conforming to recent international recommendations. J Clin Endocrinol Metab.

[53] Sowers M, Zheng H, Tomey K (2007). Changes in body composition in women over sic years at midlife: ovarian and chronological aging. J Clin Endocrinol Metab.

[54] Hameed M, Harridge SD, Goldspink G (2002). Sarcopenia and hypertrophy: a role for insulin-like growth factor-1 in aged muscle?. Exerc Sport Sci Rev.

[55] Rinaldi S, Kaaks R, Friedenreich CM, Key TJ, Travis R, Biessy C (2014). Physical activity, sex steroid, and growth factor concentrations in pre- and post-menopausal women: a cross-sectional study within the EPIC cohort. Cancer Causes Control.

[56] Villaverde Gutiérrez C, Torres Luque G, Ábalos Medina GM (2012). Argente del Castillo MJ, Guisado IM, Guisado Barrilao R et al. Influence of exercise on mood in postmenopausal women. J Clin Nurs.

